# Supplementary material for: RabbitTClust: enabling fast clustering analysis of millions of bacteria genomes with MinHash sketches
Source: Genome Biol. 2023 May 17;24:121. doi: 10.1186/s13059-023-02961-6 (PMC10190105; doi:10.1186/s13059-023-02961-6)
Supplement: Supplementary file 1 — Additional file 1: Figure S1. The performance evaluation of RabbitTClust on a 64-core Intel workstation. [file 13059_2023_2961_MOESM1_ESM.pdf]

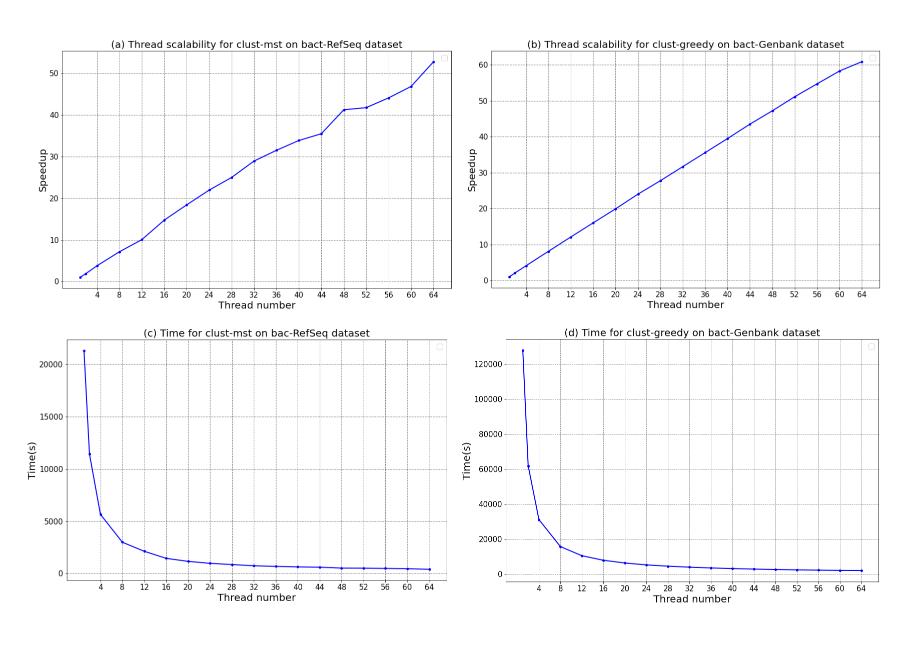

Figure S1: Thread scalability for *clust-mst* and *clust-greedy* on a 64-core Intel workstation (two 32-core Xeon platinum 8375C CPUs, 256 GB DDR4 RAM, 7.68 TB WD SN640 SSD). Using 64 threads, *clust-mst* completed clustering of *bact-RefSeq* in just 7 minutes with a memory footprint of 6.79 GB. Similarly, *clust-greedy* completed clustering of *bact-GenBank* in 38 minutes using 64 threads, with a memory footprint of 15.57 GB.
