# Supplementary material for: RabbitTClust: enabling fast clustering analysis of millions of bacteria genomes with MinHash sketches
Source: Genome Biol. 2023 May 17;24:121. doi: 10.1186/s13059-023-02961-6 (PMC10190105; doi:10.1186/s13059-023-02961-6)
Supplement: Supplementary file 5 — Additional file 5: Review history. [file 13059_2023_2961_MOESM5_ESM.docx]

Review History

**First round of review**

**Reviewer 1**

**Were you able to assess all statistics in the manuscript, including the appropriateness of statistical tests used?** There are no statistics in the manuscript.

**Were you able to directly test the methods?** No.

**Comments to author:**

This paper presents RabbitTClust, a novel tool designed to cluster large-scale genomic data through the utilization of sketch-based distance estimation. The tool employs a combination of streaming and parallelization techniques, which effectively reduce memory usage and improve runtime efficiency. RabbitTClust provides users with two sub-commands, clust-mst and clust-greedy, that can be used in different scenarios.

The experimental results show that RabbitTClust can cluster more than one million bacterial genomes within a few minutes on a 128-core workstation. Additionally, the tool can identify redundant genomes within the NCBI RefSeq and GenBank databases. The proposed method is sound, and the paper is well-written and easy to follow. RabbitTClust is helpful in advancing the scalability of bioinformatics methods to ever-increasing dataset sizes.

Comments:

1. What's the detailed configuration of the 128-core workstation (Intel or AMD CPUs)? The author should test the performance of RabbitTClust on both Intel and AMD platforms.

2. On page 4, line 22 and line 23, the scripts should be marked in italics.

3. The font size of the text in Figure 6 is too small. It makes the Figure hard to read, especially when the document is printed.

**Reviewer 2**

**Were you able to assess all statistics in the manuscript, including the appropriateness of statistical tests used?** Yes, and I have assessed the statistics in my report.

**Were you able to directly test the methods?** Yes.

**Comments to author:**

The paper proposes a software tool for clustering genomic sequences, typically a large number of bacterial genomes. The tools contains two modules, called clust-mst and clust-greedy, implementing two differents clustering strategies. The paper reports successful large-scale experiments on clustering order of 10^5-10^6 bacterial genomes from RefSeq and GenBank.

On the methodological/algorithmic side, the papet brings no novelty. The software implements a combination of existing algorithms such as MinHash sketching (similarity and containment versions), MST-based hierarchical clustering, greedy incremental clustering, known criteria for clustering quality (NMI), etc.

On the biological side, the contribution is small as well, as the authors don't carry much analysis of their experiments as far as biological outcomes are concerned. The only exception here is the Section "Filtering redundant genomes" where the authors do some analysis of redundancy and mislabeling occurring in RefSeq and GenBank databases.

Thus, the contribution of the work is mainly in software developement, where the authors performed a significant engineering work on boosting the software efficiency by employing software optimization techniques such as multi-threading, SIMD vectorization, etc. As a result, they were able to carry out terabyte-scale experiments on a computer workstation. This is a significant achievement which shows the feasibility of such a computation and demonstrates the usefulness of the tool.

Still, I consider that the contribution may not be innovative enough for Genome Biology and may be more suitable for a more bioinformatics-oriented venue.

The paper is clearly written and easy to follow. In my view, the presentation is somewhat "slow" with some points repeated or too elementary to be explained in detail in a paper of this level. This is a minor issue, however.

**Authors Response**

**Point-by-point responses to the reviewers’ comments:**

Reviewer: 1

This paper presents RabbitTClust, a novel tool designed to cluster large-scale genomic data through the utilization of sketch-based distance estimation. The tool employs a combination of streaming and parallelization techniques, which effectively reduce memory usage and improve runtime efficiency. RabbitTClust provides users with two sub-commands, clust-mst and clust-greedy, that can be used in different scenarios.

The experimental results show that RabbitTClust can cluster more than one million bacterial genomes within a few minutes on a 128-core workstation. Additionally, the tool can identify redundant genomes within the NCBI RefSeq and GenBank databases. The proposed method is sound, and the paper is well-written and easy to follow. RabbitTClust is helpful in advancing the scalability of bioinformatics methods to ever-increasing dataset sizes.

***Response:***

***We appreciate the positive feedback.***

Comments:

1. What’s the detailed configuration of the 128-core workstation (Intel or AMD CPUs)? The author should test the performance of RabbitTClust on both Intel and AMD platforms.

***Response:***

***The AMD workstation utilized in this study is equipped with two 64-core AMD EPYC 7T83 CPUs, 256 GB DDR4 RAM, and a 7.68 TB WD SN640 SSD.***

***The detailed specifications have been included in the manuscript. Moreover, we evaluated the performance of RabbitTClust on a 64-core Intel workstation comprising dual 32-core Xeon platinum 8375C CPUs, 256 GB DDR4 RAM, and a 7.68 TB WD SN640 SSD. As demonstrated in Additional file 1, RabbitTClust demonstrates similar scaling performance on the Intel platform.***

2. On page 4, line 22 and line 23, the scripts should be marked in italics.

***Response:***

***We have marked the scripts in italics.***

3. The font size of the text in Figure 6 is too small. It makes the Figure hard to read, especially when the document is printed.

***Response:***

***We have updated Figure 6 with a larger font size.***

Reviewer: 2

The paper proposes a software tool for clustering genomic sequences, typically a large number of bacterial genomes. The tools contains two modules, called clust-mst and clust-greedy, implementing two differents clustering strategies. The paper reports successful large-scale experiments on clustering order of 10^5-10^6 bacterial genomes from RefSeq and GenBank.

On the methodological/algorithmic side, the papet brings no novelty. The software implements a combination of existing algorithms such as MinHash sketching (similarity and containment versions), MST-based hierarchical clustering, greedy incremental clustering, known criteria for clustering quality (NMI), etc. On the biological side, the contribution is small as well, as the authors don’t carry much analysis of their experiments as far as biological outcomes are concerned. The only exception here is the Section “Filtering redundant genomes” where the authors do some analysis of redundancy and mislabeling occurring in RefSeq and GenBank databases.

Thus, the contribution of the work is mainly in software developement, where the authors performed a significant engineering work on boosting the software efficiency by employing software optimization techniques such as multi-threading, SIMD vectorization, etc. As a result, they were able to carry out terabyte-scale experiments on a computer workstation. This is a significant achievement which shows the feasibility of such a computation and demonstrates the usefulness of the tool.

Still, I consider that the contribution may not be innovative enough for Genome Biology and may be more suitable for a more bioinformatics-oriented venue.

The paper is clearly written and easy to follow. In my view, the presentation is somewhat “slow” with some points repeated or too elementary to be explained in detail in a paper of this level. This is a minor issue, however.

***Response:***

***We thank the reviewer for the valuable comments. In order to avoid redundancy, we have eliminated the repeated RabbitTClust efficiency description in the “Background” section, as it has been presented in detail in the “Results” section. Furthermore, we have changed the name of the “RabbitTClust overview” subsection to “Performance Comparison” in the “Results” section, where the repeated description of the RabbitTClust pipeline has also been removed. In addition, we have removed the duplicated definitions of Mash distance and AAF distance in the “Sketching and Distance Measurement” and “Distance Threshold” subsections. To make the presentation less “slow”, certain parts such as the parameter analysis have been shortened without compromising the essential details.***
